# Antioxidant, Anti-Nephrolithe Activities and *in Vitro* Digestibility Studies of Three Different Cyanobacterial Pigment Extracts

**DOI:** 10.3390/md13085384

**Published:** 2015-08-20

**Authors:** Chetan Paliwal, Tonmoy Ghosh, Khushbu Bhayani, Rahulkumar Maurya, Sandhya Mishra

**Affiliations:** 1 Salt and Marine Chemicals, CSIR-Central Salt and Marine Chemicals Research Institute, Bhavnagar 364002, India; E-Mails: paliwalchetan@gmail.com (C.P.); forghosh@gmail.com (T.G.); khushboobhayani@gmail.com (K.B.); maurya.micro@gmail.com (R.M.); 2 Academy of Scientific & Innovative Research (AcSIR), CSIR-Central Salt and Marine Chemicals Research Institute, Bhavnagar 364002, India

**Keywords:** Carotenoids, phycobiliproteins, Antioxidants, iso-bolographic analysis, calcium oxalate crystallization, *in vitro* digestibility, DPPH

## Abstract

Phycobiliprotein-containing water and carotenoid-containing methanolic extracts of three different cyanobacteria, *Pseudanabaena* sp., *Spirulina* sp. and *Lyngbya* sp., were studied for their DPPH scavenging, iso-bolographic studies, and anti-nephrolithe activities. The best EC_50_ values for DPPH scavenging were in *Lyngbya* water (LW, 18.78 ± 1.57 mg·mg^−1^ DPPH) and *Lyngbya* methanol (LM, 59.56 ± 37.38 mg·mg^−1^ DPPH) extracts. Iso-bolographic analysis revealed most of the combinations of extracts were antagonistic to each other, although LM—*Spirulina* methanol (SM) 1:1 had the highest synergistic rate of 86.65%. *In vitro* digestion studies showed that DPPH scavenging activity was considerably decreased in all extracts except for *Pseudanabaena* methanol (PM) and LM after the simulated digestion. All of the extracts were effective in reducing the calcium oxalate crystal size by nearly 60%–65% compared to negative control, while PM and *Spirulina* water (SW) extracts could inhibit both nucleation and aggregation of calcium oxalate by nearly 60%–80%.

## 1. Introduction

Microalgae are ecumenical, photosynthetic, prokaryotic organisms found in diverse habitats and extreme environmental conditions covering both aquatic and terrestrial territories [1]. They are a source of primary productivity in an aquatic ecosystem. Estimates have calculated the productivity of microalgae within a range of one-third to more than half of the global primary productivity [2,3]. They have the capacity to adjust in varying adverse environmental conditions. Being such prolific organisms, they are a storehouse of various commercially important and beneficial compounds like phycobiliproteins, carotenoids, polysaccharides, vitamins, polyhydroxyalkanoates, and biocides [4,5,6,7,8]. Phycobiliproteins and carotenoids, respectively, are water- and lipid-soluble pigments, polysaccharides are complex carbohydrates which can be used as emulsifiers, polyhydroxyalkanoates are a group of compounds which can be used as biodegradable plastics, while biocides are anti-bacterial, anti-fungal, anti-viral or even anti-algal compounds. There is a huge market demand for these important bioactive compounds, which has provided a thrust in their research and development [9]. Among these, water-soluble phycobiliproteins and lipo-soluble carotenoids play a key role in light harvesting and protection from photo-oxidative stress. These compounds have also been center of attraction due to their natural antioxidant potential with outstanding properties to fight against oxidative stress, cancer, aging, and other degenerative diseases [10,11,12].

Urolithiasis has been a frequently seen problem in humans and it affects a large section of the human population. In more than 80% of the cases these are calcium rich stones formulated by either oxalate or phosphate. Crystallization of these stones is affected by various physicochemical events, such as nucleation, growth, and aggregation. Recent studies suggest that antioxidants and free radical scavengers have an ability to counter the occurrence and reoccurrence of urinary stones [13].

The integration of these biologically-active pigments in food products would increase their nutritional and health benefits. Recently, great interest has developed in the *in vitro* model for food delivery systems to study the release of desired compounds that will benefit human health [14]. *In vitro* digestion processes are widely used to gain an insight into the gastrointestinal changes that a food will undergo; for example, structural changes and release of specific components, under controlled gastrointestinal conditions. *In vitro* digestion methods have advantages over *in vivo* methods because of their rapidity, economics, labor requirements, and lack of any ethical issues. There have been several *in vitro* digestibility studies carried out on variety of natural compounds to assess their bioavailability but studies have been lacking on pigment extracts of cyanobacteria, although there have been *in vitro* digestibility studies on synthetic carotenoids absorption in humans [15]. This method provides an acceptable validation of the digestive process in comparison with *in vivo* tests [16]. 

The aim of this study is to investigate the free radical scavenging activities of water and methanolic extracts of three chosen cyanobacterial strains and also to observe the synergistic effects when these extracts are mixed with each other in different proportions. It also aims to conduct studies on the *in vitro* digestibility of the said extracts and to evaluate their potential for free radical scavenging after the digestion process. This study would enhance our current knowledge about the behavior of cyanobacterial pigments and their antioxidant activities before and after digestion. Further, it also aims to investigate the effects of these extracts on the inhibition of calcium oxalate crystallization, the most common cause of urolithiasis.

## 2. Results and Discussion

### 2.1. Characterization of Extracts

The extracts were characterized as shown in Section 3.2. The phycobiliprotein profile of water and carotenoid profile of methanolic extracts have been presented in Table 1. The *Spirulina* water (SW) extract had C-phycocyanin (C-PC) as the major pigment (11.62 ± 0.30 mg·g^−1^). Similarly, the *Spirulina* methanolic (SM) extract had echinenone as the major carotenoid (0.38 ± 0.04 mg·g^−1^) with a β-carotene content of 0.23 ± 0.02 mg·g^−1^. The *Pseudanabaena* water (PW) extract had C-phycoerythrin (C-PE) as the major pigment (9.45 ± 0.45 mg·g^−1^), while its methanolic (PM) extract mainly contained zeaxanthin (0.29 ± 0.02 mg·g^−1^). *Lyngbya* water (LW) extract had C-PE as the major pigment (108.76 ± 2.18 mg·g^−1^), while its methanolic (LM) extract mainly contained β-carotene (0.17 ± 0.03 mg·g^−1^). All the values have been reported in triplicate on a dry mass basis.

**Table 1:** Phycobiliprotein and carotenoid profile of water and methanolic extracts, respectively, for different cyanobacterial species (mg·g^−1^ dry mass, *n* = 3).

Compounds (mg·g^−1^ dry mass)		*Pseudanabaena* sp.	*Spirulina* sp.	*Lyngbya* sp.
Phycobiliproteins (Water extract)	C-PC ^a^	6.96 ± 0.34	11.62 ± 0.3	107.92 ± 1.79
	A-PC ^a^	2.94 ± 0.2	3.87 ± 0.11	21.64 ± 0.75
	C-PE ^a^	9.45 ± 0.45	0.93 ± 0.02	108.76 ± 2.18
Carotenoids (Methanol extract)	Myxoxanthophyll	0.05 ± 0.01	0.02 ± 0.00	0.05 ± 0.02
	Zeaxanthin	0.29 ± 0.02	0.12 ± 0.01	0.15 ± 0.13
	Canthaxanthin	0.11 ± 0.01	0.04 ± 0.00	0.04 ± 0.01
	Lycopene	0.12 ± 0.01	0.17 ± 0.02	ND ^a^
	α-carotene	0.08 ± 0.00	0.09 ± 0.01	0.10 ± 0.02
	β-carotene	0.27 ± 0.02	0.23 ± 0.02	0.17 ± 0.03
	Echinenone	ND ^a^	0.38 ± 0.04	ND ^a^
Chlorophyll (Methanol extract)	Chlorophyll a	0.70 ± 0.03	4.80 ± 0.51	1.5 ± 0.03

^a^ C-PC: C-phycocyanin, A-PC: allophycocyanin, C-PE: C-phycoerythrin, ND: not detected.

Morist *et al.* have reported C-PC content of 40 mg·g^−1^ (w/w) in *Spirulina platensis* cultured in a photobioreactor, while Mishra *et al.* have reported a C-PE content of 25.6 mg·g^−1^ in *Pseudanabaena* sp. after 60% ammonium sulphate fractionation [17,18]. In another reported study, *Anabaena* sp. was reported to contain approximately 83 mg·g^−1^ of C-PE [19].

If we compare the lipophilic carotenoids in these three species with earlier reported values, Aakermann *et al.* have reported 1.3 mg·g^−1^ total carotenoids in *Spirulina platensis* out of which 0.46 mg was β-carotene and 0.03 mg was echinenone. They have also reported similar figures for echinenone and β-carotene in another *Spirulina subsalsa* strain [20]. Singh has reported total carotenoid content ranging from 0.18 ± 0.00 to 9.74 ± 0.53 mg·g^−1^ of several different *Lyngbya* sp. strains [21]. They have also reported the total carotenoid content in three *Pseudanabaena* species in a range of 4.44 ± 0.67 to 6.64 ± 0.28 mg·g^−1^.

### 2.2. Antioxidant Activities

#### 2.2.1.Total Phenolic Content (TPC)

**Figure 1:**
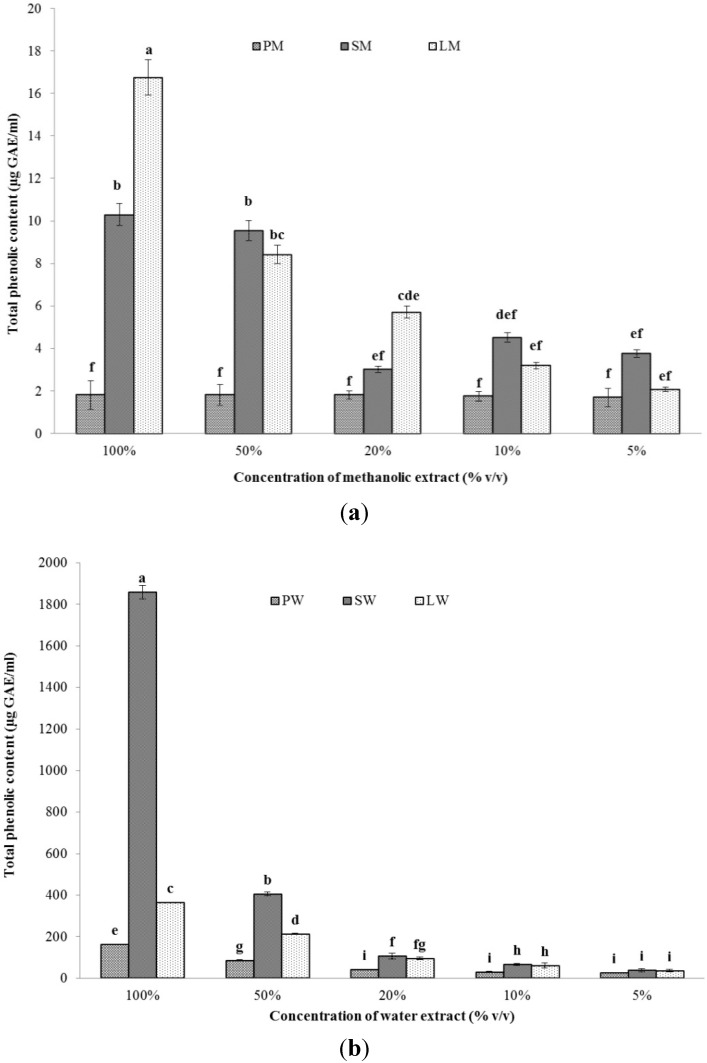
Total phenolic content of (**a**) methanolic and (**b**) water extracts of *Pseudanabaena* sp. (PM, PW), *Lyngbya* sp. (LM, LW), and *Spirulina* sp. (SM, SW) at different concentration (*n* = 3).

The total phenolic content of the methanol and water extracts have been shown in Figure 1a,b, respectively. The extracts were diluted to five different concentrations which were then evaluated according to the method detailed in Section 3.3.1. The phenolic content decreased with the dilution of the extracts. In case of water extracts, the highest phenolic content was seen in LW extract (278.73 ± 1.24 mg gallic acid equivalent (GAE) g^−1^ dry mass), followed by SW extract (119.19 ± 2.09 mg GAE g^−1^ dry mass). The same trend was observed when the methanolic extracts were considered. The highest phenolic content was observed in LM extract (12.77 ± 2.23 mg GAE g^−1^ dry mass), with SM extract placing second (0.66 ± 0.3 mg GAE g^−1^ dry mass). The PW and PM extracts contained the lowest amount of phenolic content. All the values have been presented in triplicate. Jiang *et al.* have reported phenolic contents of four different vegetable extracts in a range of 0.64 ± 0.04 to 8.88 ± 0.59 mg GAE g^−1^ dry mass [22]. Machu *et al.* have reported phenolic contents of 43.2 ± 1.0 mg GAE g^−1^ from edible dried *Spirulina platensis* when extracted in heated distilled water [23]. When using 100% methanol, the corresponding value is 24.4 ± 0.2 mg GAE g^−1^. However, we have utilized the same biomass for both water and methanol extraction, which accounts for the lower phenolic content in the methanol extracts. Ismaiel *et al.* have reported phenolic contents of 1–10 mg GAE g^−1^ dry mass from the water extracts of eleven different cyanobacterial strains with the highest content in *Spirulina platensis* [24]. In the present study, both LW and SW can contribute to various biological activities due to the considerably higher phenolic content. These phenolics are thought to play an important role in the antioxidant activity of the respective extracts by donating electrons. However, the total phenolic content of an extract is often more meaningful, both scientifically and economically, instead of extracting and purifying different phenolic compounds individually, since they may act co-operatively with each other rather than alone [25].

#### 2.2.2. Total Antioxidant Capacity (TAC)

**Figure 2:**
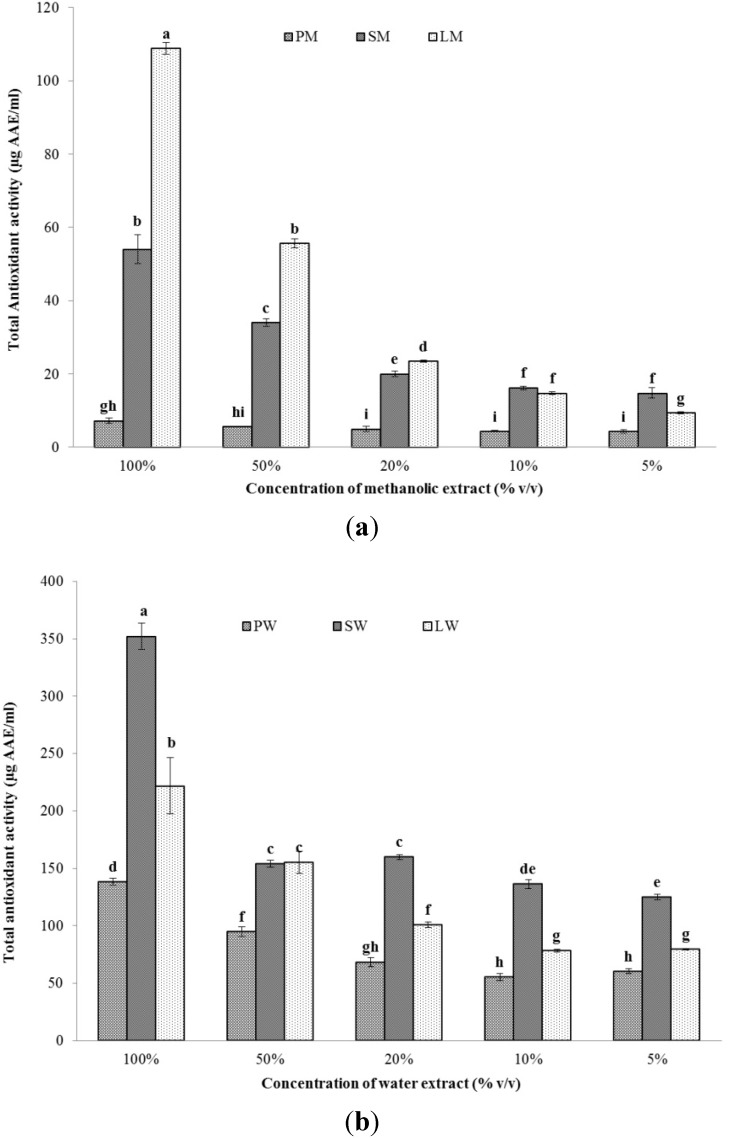
Total antioxidant capacity of (**a**) methanolic and (**b**) water extracts of *Pseudanabaena* sp. (PM, PW), *Lyngbya* sp. (LM, LW), and *Spirulina* sp. (SM, SW) at different concentrations (*n* = 3).

The total antioxidant capacity of the methanol and water extracts has been shown in Figure 2a,b, respectively. The native extracts were, again, diluted to get five different concentrations of the extracts. Similar to total phenolic content, water extracts LW and SW, again, showed a higher antioxidant activity. LW had an activity of 169.42 ± 18.54 mg ascorbic acid equivalents (AAE) g^−1^ dry mass. However, the anti-oxidant capacity of PW was higher than SW in this test (49.92 ± 1.11 mg AAE g^−1^ dry mass). In the case of the methanolic extracts, LM had the highest activity (83.11 ± 1.19 mg AAE g^−1^ dry mass), followed by SM (3.46 ± 0.36 mg AAE g^−1^ dry mass), while PM, again, had the lowest activity of all the three extracts. The priority of water extraction over methanol, again, led to a higher activity in water extracts. Ganesan *et al.* have reported anti-oxidant capacities of extracts from Indian red seaweeds ranging from 0.31 ± 0.1 to 2.88 ± 0.39 mg AAE g^−1^ extract [26]. Additionally, Kumar *et al.* have studied the antioxidant capacities of 22 tropical seaweeds from India and found them within a range of 0.21 ± 0.06 to 1.14 ± 0.16 mg AAE g^−1^ extract [27]. Considering the significantly higher activities, the cyanobacterial extracts can be considered valuable antioxidants.

#### 2.2.3. DPPH Scavenging Activity and EC_50_ Value

The water and methanolic extracts were evaluated for their 2,2-diphenyl-1-picrylhydrazyl (DPPH) scavenging activity and the EC_50_ values were calculated on a dry mass basis. DPPH is a common free radical used to assess the radical scavenging activity of many extracts and antioxidants [28,29]. EC_50_ typically represents the amount of a compound needed to reduce the concentration of the free radical by half. A lower EC_50_ value signifies a better scavenging activity. The DPPH scavenging activity of the native extracts, as well as the corresponding EC_50_ value, has been shown in Table 2. LW was found to be the best scavenger of DPPH with an EC_50_ value of 18.78 ± 1.57 mg dry mass mg^−1^ DPPH, while LM was the best scavenger in case of methanolic extracts (EC_50_ of 59.56 ± 37.38 mg dry mass mg^−1^ DPPH). The scavenging effect of both water and methanolic extracts shows that the antioxidant activity of the extracts is mainly due to the presence of pigments in them.

**Table 2:** TPC, TAC, and antioxidant activities of different cyanobacterial extracts (*n* = 3).

Sample	TPC (mg GAE g^−1^ dry mass)	TAC (mg AAE g^−1^ dry mass)	EC_50_ DPPH (mg dry mass mg^−1^ DPPH)
PW	58.94 ± 0.34	49.92 ± 1.11	42.73 ± 4.29
SW	119.19 ± 2.09	22.59 ± 0.74	148.46 ± 21.17
LW	278.73 ± 1.24	169.42 ± 18.54	18.78 ± 1.57
PM	0.65 ± 0.24	2.61 ± 0.27	1544.43 ± 207.76
SM	0.66 ± 0.3	3.46 ± 0.26	728.55 ± 667.73
LM	12.77 ± 2.23	83.11 ± 1.19	59.56 ± 37.38

### 2.3. Iso-Bolographic Studies

The interaction of different extracts with each other and their effects on DPPH scavenging were studied using iso-bolographic studies (Table 3). Most of the extracts were antagonistic to each other with the theoretical sum of their EC_50_ values less than their corresponding experimental values. Synergistic effects were observed for the combinations of LM-SM, SM-PM, and SM-PW only. The descending order of synergy between different combinations were LM-SM 1:1 (86.65%) > SM-PW 9:1 (83.18) > SM-PW 1:1 (82.2%) > LM-SM 1:9 (75.18%) > LW-SW 1:1 (64.6%) > LM-SM 9:1 (57.6%) > SW-PM 1:9 (48.88%) > SM-PM 9:1 (42.74%). The antagonistic effects observed in other combinations may be due to other proteins or compounds present in the water extracts, since most of the methanolic extracts have excellent synergy between them. Cyanobacteria have been reported to secrete various toxins that might have played a role during the combination of different extracts [30,31]. Little data is available on iso-bolographic studies conducted on plant extracts since most of the studies have been conducted on drug interactions. Jiang *et al.* have reported the use of iso-bolography for evaluating the synergistic and antagonistic effects of combinations of hydrophilic and lipophilic extracts of four vegetables [22]. They have reported that most of the combinations have high levels of synergy between them, with the lipophilic carrot-hydrophilic eggplant 1:1 combination having 87.4% synergistic rate, which is similar to our observation for LM-SM 1:1.

**Table 3:** DPPH activities of different cyanobacterial extracts in combination (*n* = 3) *.

Combination of Extracts	Fraction	Theoretical EC_50_ (mg mg^−1^ DPPH)	Experimental EC_50_ (mg mg^−1^ DPPH)	Synergistic Rate (%)
LW-PW	F1/9	40.33	185.21 ± 9.23 ^a^	−359.20
	F5/5	30.75	55.88 ± 22.17 ^a^	−81.71
	F9/1	21.17	26.96 ± 11.99 ^a^	−27.36
PW-SW	F1/9	137.89	261.88 ± 111.67 ^a^	−89.92
	F5/5	95.59	130.93 ± 55.45 ^a,b^	−36.97
	F9/1	53.30	88.15 ± 63.01 ^b^	−65.38
LW-SW	F1/9	135.49	242.33 ± 120.34 ^a^	−78.85
	F5/5	83.62	29.60 ± 5.18 ^b^	64.60
	F9/1	31.75	32.34 ± 17.66 ^b^	−1.88
LM-SM	F1/9	661.65	164.23 ± 84.79 ^a^	75.18
	F5/5	394.06	52.60 ± 17.10 ^a^	86.65
	F9/1	126.46	53.63 ± 30.31 ^a^	57.60
LM-PM	F1/9	1395.94	5371.82 ± 628.95 ^a^	−284.82
	F5/5	802.00	883.11 ± 89.96 ^a^	−10.11
	F9/1	208.05	1388.47 ± 320.20 ^a^	−567.37
SM-PM	F1/9	1462.84	1141.63 ± 427.18 ^a^	21.96
	F5/5	1136.49	726.50 ± 58.38 ^a^	36.07
	F9/1	810.14	463.86 ± 82.31 ^a^	42.74
LW-SM	F1/9	657.57	587.30 ± 93.65 ^a^	10.69
	F5/5	373.66	595.09 ± 112.33 ^a^	−59.26
	F9/1	89.75	71.10 ± 52.77 ^a^	20.79
SW-PM	F1/9	1404.83	718.10 ± 82.43 ^a^	48.88
	F5/5	846.45	1242.09 ± 204.02 ^a^	−46.74
	F9/1	288.06	450.80 ± 198.58 ^a^	−56.50
PW-LM	F1/9	57.88	50.28 ± 23.22 ^a^	13.13
	F5/5	51.15	176.53 ± 26.72 ^a^	−245.15
	F9/1	44.41	53.35 ± 18.67 ^a^	−20.13
LM-SW	F1/9	139.57	171.74 ± 74.87 ^a^	−23.05
	F5/5	104.01	690.94 ± 57.20 ^a,b^	−564.29
	F9/1	68.45	70.23 ± 13.17 ^b^	−2.60
PM-LW	F1/9	171.34	220.12 ± 45.49 ^a^	−28.47
	F5/5	781.60	858.59 ± 166.33 ^a^	−9.85
	F9/1	1391.87	1908.85 ± 388.08 ^a^	−37.14
SM-PW	F1/9	111.31	94.51 ± 51.36 ^a^	15.09
	F5/5	385.64	68.65 ± 25.04 ^a^	82.20
	F9/1	659.97	111.03 ± 78.97 ^a^	83.18

* Values with different letters (a, b) in the same column show significant difference (*p* < 0.05).

### 2.4. In Vitro Digestibility Studies

**Figure 3:**
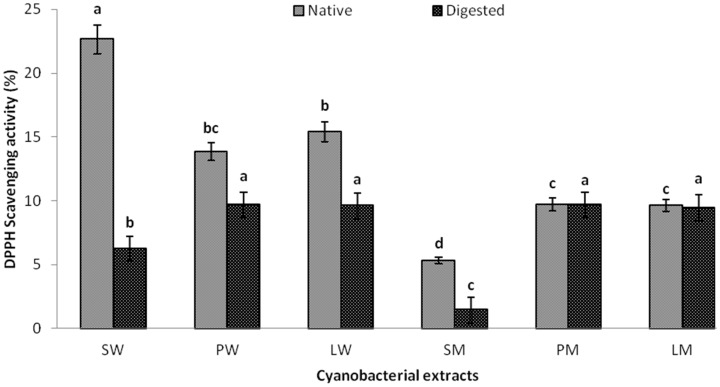
DPPH scavenging activity of water and methanolic extracts of *Pseudanabaena* sp., *Lyngbya* sp. and *Spirulina* sp. before and after digestion.

*In vitro* digestion mimics the enzymes and their optimum physico-chemical conditions found in our gastro-intestinal tract. It provides an excellent alternate to animal feeding experiments to observe the changes taking place in a food item after ingestion. We investigated the effect of simulated digestion on the cyanobacterial extracts according to the protocol detailed in Section 3.5. All of the water extracts showed reduced DPPH scavenging after digestion (Figure 3). The probable reason would be the degradation of the amino acid backbone of the proteins in the water extract due to the presence of pepsin in gastric and pancreatin in duodenal solution. To the best of our knowledge, no studies on *in vitro* digestion of cyanobacterial extracts containing phycobiliproteins and carotenoids have been reported, which is an excellent avenue for further research. However, there is a recent report of R-phycoerythrin (R-PE) from *Bangia fusco-purpurea* that gave higher values of DPPH radical scavenging activities after digestion. The reason was the formation of short peptides after digestion in which the hydrophobic amino acid residues were more exposed to DPPH radical and contributed to the overall anti-oxidant activity [32]. Among the methanol extracts SM showed a considerable decrease in the scavenging activity while PM and LM were relatively unchanged. Though no study has reported the *in vitro* digestibility studies of methanolic extracts of microalgae, the digestion of β-carotene alone has previously been carried out. According to reports on human digestion studies, β-carotene absorption is highly variable and inefficient due to their high hydrophobicity and consequently, less solubility in aqueous systems [15]. This is the probable reason for the unchanged activities of methanolic extracts of PM and LM.

### 2.5. Effect of Water and Methanolic Extracts on Calcium Oxalate Crystallization

**Figure 4:**
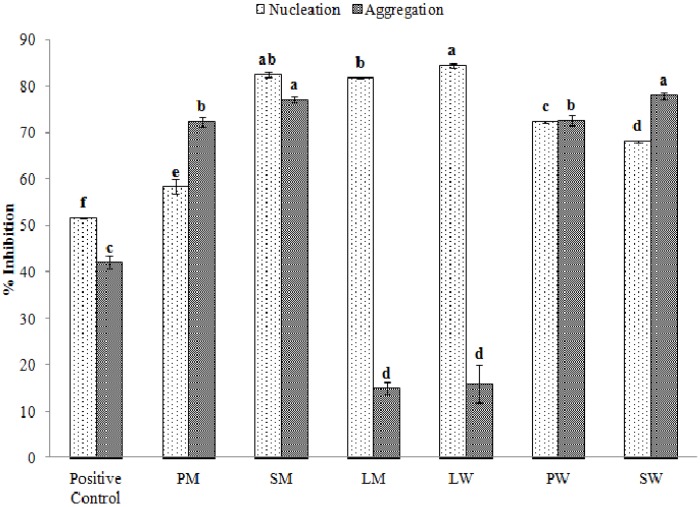
% inhibition of nucleation and aggregation of calcium oxalate crystals by different extracts (PM: *Pseudanabaena* sp. methanol extract, SM: *Spirulina* sp. methanol extract, LM: *Lyngbya* sp. methanol extract, LW: *Lyngbya* sp. water extract, PW: *Pseudanabaena* sp. water extract, SW: *Spirulina* sp. water extract).

The formation of calcium oxalate and other types of crystals is a common cause of urolithiasis, more commonly known as urinary stones. Approximately 10% of the world’s population is affected by it, out of which around 90% of the cases are related to the formation of calcium oxalate crystals. There are currently no drugs available for its treatment. The supersaturation of urine by calcium oxalate is a central process to the stone formation activity in which two separate processes, crystal nucleation, and crystal aggregation, are involved. When the supersaturation exceeds the limit of metastability, calcium and oxalate form clusters that are the core of the crystals (nucleation). The addition of new ions to this core causes the crystals to grow in size, after which they start merging into one another to form the macroscopic stones (aggregation). The processes of nucleation and aggregation can be evaluated through observing the kinetics of a metastable solution of calcium and oxalate maintained at physiological pH-value and temperature. It is also possible to calculate the rates of nucleation and aggregation through observing the optical density of the solution at 620 nm. Figure 4 shows the rates of inhibition of nucleation and aggregation of calcium oxalate in the presence or absence of our test samples compared to the negative control. The cyanobacterial water extracts were able to inhibit the nucleation and aggregation processes up to 84.43% ± 0.44% and 77.97% ± 0.66%, respectively. The corresponding values in the methanolic extracts were 82.49% ± 0.55% and 77.10% ± 0.66%. Tri-sodium citrate was taken as the positive control and inhibited the nucleation and aggregation processes by 51.52% ± 0.01% and 42.03% ± 1.33%, respectively. Different extracts used in our study influenced the crystallization process in different ways. For example, the extracts of *Lyngbya* sp. inhibit the process of nucleation significantly more than they inhibit aggregation. Overall, the PW and SM extracts inhibit both nucleation and aggregation almost equally and are the optimum choices (Supplementary Table 1). There are no studies available on the calcium oxalate crystallization assay of microalgal extracts whereas Teodesio Melo *et al.*, have investigated the anti-stone activity of sulphated polysaccharides from brown seaweed *Dictyopteris justii* wherein they have obtained inhibition rates of close to 80%–85% for both nucleation and aggregation [33]. Similar rates for both these processes were observed in our native extracts. Further, Zhang *et al.* have reported similar values of inhibition of nucleation and aggregation when they utilized sulphated polysaccharides from another brown seaweed, *Sargassum graminifolium* [13].

**Figure 5:**
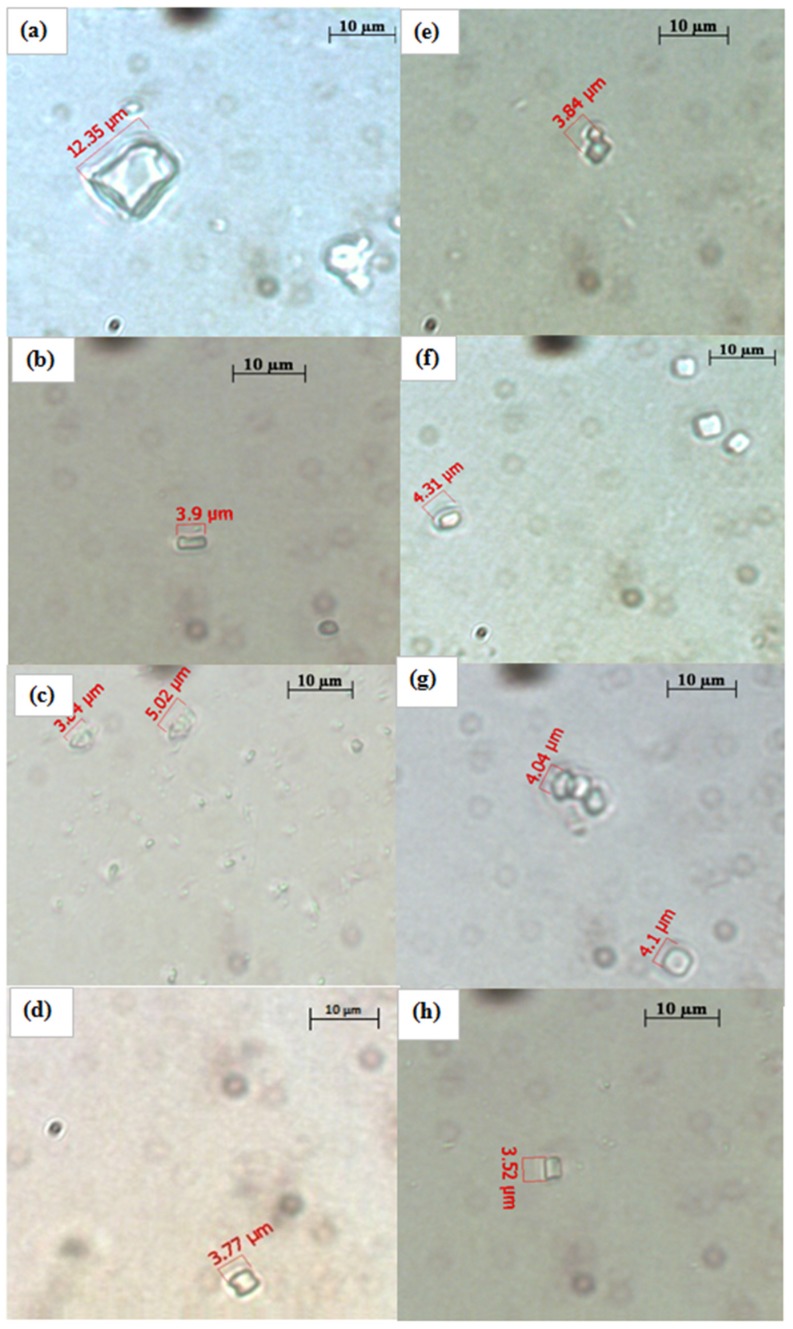
CaOx crystals observed under inverted microscope (100×) formed in the metastable solution of CaOx in the (**a**) absence of sample and the presence of (**b**) PM; (**c**) SM; (**d**) LM; (**e**) trisodium citrate; (**f**) PW; (**g**) SW; (**h**) LW.

The morphology of the crystals have been shown in Figure 5a–h. The crystal size in the negative control is approximately 12 μm in size (Figure 5a) while the test samples (Figure 5b–h) were able to reduce the size of the crystals by almost 60%–65% (approximately 3–4 μm). Such types of crystals can be more easily excreted out from the body during the passage of urine. The observation is also supported by the significant inhibition of nucleation and aggregation processes (Figure 4).

## 3. Experimental Section 

### 3.1. Preparation of Extracts

*Spirulina* sp. was grown in Zarrouk’s medium, while *Pseudanabaena* sp. and *Lyngbya* sp. were both grown in ASN III medium according to earlier published reports [18,34]. The biomass was harvested after 18 days through centrifugation (10,000× *g*, 10 min, 10 °C, Kubota Corporation, Japan) and washed thoroughly with distilled water to remove salt and particulates. The wet biomass was directly taken for extraction of pigments.

Water extracts: The harvested biomass was freeze thawed repeatedly in distilled water (−70 °C followed by 25 °C). After approximately 5–6 cycles of freeze thaw, the extract was centrifuged (10,000× *g*, 10 min, 4 °C) and was stored at −80 °C until further experiments.

Methanol extracts: Pre-weighed lyophilized microalgal biomass (approximately 100–200 mg) left after the water extraction process was dissolved in 5 mL of 99.9% pure methanol and vortexed thoroughly to increase the solvent contact time for maximum carotenoid extraction. The suspensions were incubated at 45 °C for 24 h in the dark, as described in Pancha *et al.* [35]. After 24 h, the extract was centrifuged at 10,000× *g* for 5 min (4 °C) to remove the cell debris and the supernatants were stored at −80 °C until further experiments.

### 3.2. Characterization of Water and Methanol Extracts

The spectra of the water extracts were recorded from 250 to 700 nm in a microplate reader (SpectraMAX 190, Molecular Devices Inc., Sunnyvale, CA, USA). The concentrations of C-PC, Allophycocyanin (APC) and C-PE were calculated using the equations of Bennett and Bogorad [36]:

(1) $$\text{C-PC}\;(\text{mg}\cdot {\text{mL}}^{-1})=[{\text{A}}_{615}-0.474{\text{A}}_{652}]/5.34$$

(2) $$\text{A-PC}\;(\text{mg}\cdot {\text{mL}}^{-1})=[{\text{A}}_{652}-0.208{\text{A}}_{615}]/5.09$$

(3) $$\text{C-PE}\;(\text{mg}\cdot {\text{mL}}^{-1})=[{\text{A}}_{562}-2.41(\text{C-PC})-0.849(\text{A-PC})]/9.62$$

The methanol extracts were analyzed through a Shimadzu HPLC system equipped with a diode array detector using a modified method described by Anderrson *et al.* [37]. The samples were eluted with a binary solvent gradient with Solvent A (v/v), a mixture of 80% acetonitrile, 15% MeOH and 5% dichloromethane and Solvent B (v/v), a mixture of 30% acetonitrile, 20% MeOH and 50% dichloromethane. The flow rate was 1 ml/min with the gradient flow of 0% B (0–2 min), 0%–25% B (2–15 min), 25%–60% B (15–17 min), 60%–90% B (17–29 min), 90% B (29–39 min), 90%–0% B (39–41 min), and 0% B (41–47 min) in a 4.6 × 250 mm TSKgel ODS 120 T column (Tosoh Corporation, Japan). Samples were analyzed using the retention times of carotenoid and chlorophyll standards obtained from DHI, Denmark. The diode array detector was set at 437 nm and concentrations were quantified based on the standard area.

### 3.3. Antioxidant Activities

#### 3.3.1. Total Phenolic Content

The total phenolic content was measured by Folin–Ciocalteu method [38]. In detail, 1 mL of the diluted sample was mixed with 4 mL of 7.5% (w/v) freshly prepared Na_2_CO_3_ and 1 mL of 10x diluted Folin–Ciocalteu reagent (Sigma Aldrich, St Louis, USA) and incubated at room temperature for 2 h. The absorbance of the samples was read at 760 nm against a reagent blank and the phenolic content was expressed in terms of μg GAE mL^−1^. The activity was further calculated on dry mass basis.

#### 3.3.2. Total Antioxidant Activity

The total antioxidant activity of the cyanobacterial extracts were analyzed through a modified method given by Ganesan *et al.* [26]. Briefly, 1 mL of sample extract and 3 mL of phospho-molybdate reagent (1.1 M H_2_SO_4_, 30 mM NaH_2_PO_4_ and 4 mM ammonium heptamolybdate) was mixed and incubated at 95 °C for 90 min. After the reaction, the mixture was allowed to cool before their absorbance was read at 695 nm. Ascorbic acid was taken as the standard. The anti-oxidant capacities of the extracts were expressed in terms of mg AAE mL^−1^. The activity was calculated on a dry mass basis.

#### 3.3.3. DPPH Assay

The DPPH scavenging activity measurement was performed using a modification of the method described by Siler *et al.* [39]. Briefly, 1 mL of a 60 μM methanolic DPPH solution was incubated with 100 μL of the test samples or water for 30 min in the dark. The absorbance was recorded at 517 nm against a blank. The % DPPH scavenging activity was measured by using following equation:

(4) $$Scavenging\;effect=[(A_{\text{control}}-A_{\text{sample}})/A_{\text{control}}]\times 100\%$$

### 3.4. Iso-Bolographic Studies

Iso-bolographic studies of the various extracts were carried out according to the method described by Jiang *et al.*, [22]. The water and methanol extracts of all the three cyanobacterial species were mixed in three different fractions. LM-SM fraction F 1/9 signifies that 1 part of LM has been mixed with 9 parts of SM. Each of the fractions was analyzed for their DPPH scavenging activity as detailed in Section 3.4 and their experimental EC_50_. The theoretical EC_50_ values were calculated using the equation:

(5) $$\text{Theoretical}\;{\text{EC}}_{50}\;\text{value}=fA+(1-f)B$$

where A and B are the EC_50_ values of the individual extracts. The synergistic rate was calculated using the equation:

(6) $$\text{SR}=\frac{\text{TV}-\text{EV}}{\text{TV}}\times 100$$

where TV and EV are the theoretical and experimental EC50 values of free radical scavenging, respectively.

### 3.5. In Vitro Digestibility of Different Extracts

*In vitro* digestibility studies were performed on water and methanol free extracts by using a modification of the method described by Hur *et al.* [40]. 5 mL of each extract was mixed with 6 mL simulated saliva (pH-value ~6.8) and incubated at 37 °C for 5 min; thereafter, 12 mL of simulated gastric juice (pH-value ~2) was added and the solution was incubated 37 °C for 120 min. In the final step, the sample mixture was mixed with simulated intestinal juice (12 mL duodenal juice + 6 mL bile juice, pH-value 8) and was incubated at 37 °C for 120 min. The samples were shaken in an incubator shaker (Jeio Tech, South Korea) at 80 rpm throughout the experiment. 1 mL of the sample was withdrawn and the reaction was stopped by addition of 4 mL absolute ethanol. The final digested samples were stored at −80 °C for analyzing their DPPH scavenging and anti-stone activities.

### 3.6. Calcium Oxalate Crystallization Assay

The effect of native and digested extracts of the three cyanobacterial species was evaluated using the calcium oxalate crystallization assay as described by Zhang *et al.* [13]. The assay quantifies the rate of formation and aggregation of calcium oxalate crystals and the efficacy of the test substances in hindering their formation by monitoring the kinetics of a metastable solution of calcium and oxalate ions at 620 nm. The slopes used for the calculation of nucleation and aggregation rates were designated as S_N_ and S_A_, respectively. The percent inhibition for nucleation and aggregation were calculated by following equations:

(7) $$\text{Nucleation}\;\text{inhibition}\;(\%)=[1-({\text{S}}_{\text{Nm}}/{\text{S}}_{\text{Nc}})]\times 100$$

(8) $$\text{Aggregation}\;\text{inhibition}\;(\%)=[1-({\text{S}}_{\text{Am}}/{\text{S}}_{\text{Ac}})]\times 100$$

where m signifies the test material and c signifies control (*tri*-sodium citrate). The morphology and formation of crystals were observed using bright field microscopy (Carl Zeiss, Germany) at a magnification of 100×.

### 3.7. Statistical Analysis

All experiments were performed in triplicate and the results are shown in mean ± SD. Analysis of variance was conducted using Fischer LSD test (Info Stat 3.0). The differences were calculated to be significant at *p* < 0.05.

## 4. Conclusions

The water and methanolic extracts of cyanobacterial biomass were found to have good free radical scavenging activities both individually, as well as in combination with each other. The DPPH scavenging activities agreed well with the phenolic content and total antioxidant capacity of the individual extracts. However, in the iso-bolographic studies, except for a few fractions, they were mostly antagonistic to each other, signifying the fact that their antioxidant activities are optimum when they are taken individually. The complex nature of the individual extracts could play a role in this phenomenon, but it cannot be conclusively proven and needs further study. The native extracts were also found to inhibit the formation of calcium oxalate crystals in a range of 58%–84% for crystal nucleation and 15%–78% in crystal aggregation. Morphological observations of the crystals prove that the size of the crystals is reduced when the extracts, whether native or digested, are added to the solution. However, the digested water extracts displayed considerably less DPPH scavenging activity which needs to be correlated with the digestion products formed. With the exception of *Spirulina* sp., the methanol extracts show nearly identical DPPH scavenging activities both before and after digestion, suggesting that carotenoids are not majorly affected by the digestion process and their antioxidant activity stems from their assimilation.

## Supplementary Files

Supplementary File 1
