# Supplementary material for: Antioxidant, Anti-Nephrolithe Activities and in Vitro Digestibility Studies of Three Different Cyanobacterial Pigment Extracts
Source: Mar Drugs. 2015 Aug 20;13(8):5384–401. doi: 10.3390/md13085384 (PMC4557027; doi:10.3390/md13085384)
Supplement: Supplementary File 1 [file marinedrugs-13-05384-s001.docx]

**Supplementary Information**

**Table S1.** Percentage inhibition of calcium oxalate crystallization with different cyanobacterial extracts (*n* = 3).

| **Sample** | **S_N_ (x 10^−3^) (min^−1^)** | **S_A_ (x 10^−3^) (min^−1^)** | **% Inhibition Nucleation** | **% Inhibition Aggregation** |
| --- | --- | --- | --- | --- |
| Negative control | 4.29 ± 0.02 | 3.62 ± 0.03 | NA | NA |
| Positive control | 2.08 ± 0.01 | 2.10 ± 0.12 | 51.52 ± 0.01 | 42.03 ± 1.33 |
| PM | 1.84 ± 0.15 | 0.64 ± 0.02 | 58.36 ± 1.62 | 72.32 ± 0.91 |
| SM | 0.75 ± 0.02 | 0.53 ± 0.02 | 82.49 ± 0.55 | 77.10 ± 0.66 |
| LM | 0.79 ± 0.02 | 1.33 ± 0.03 | 81.77 ± 0.17 | 14.93 ± 1.33 |
| LW | 0.67 ± 0.02 | 1.90 ± 0.09 | 84.43 ± 0.44 | 15.94 ± 4.04 |
| PW | 1.19 ± 0.02 | 0.63 ± 0.03 | 72.31 ± 0.25 | 72.61 ± 1.15 |
| SW | 1.38 ± 0.02 | 0.50 ± 0.02 | 68.09 ± 0.32 | 77.97 ± 0.66 |
